# Chronology of Anæsthesia

**Published:** 1868

**Authors:** 


### CHRONOLOGY OF ANÆSTHESIA.

The following is a chronological history of painless surgical operations during the anæsthetic stato, induced by the inhalation of narcotic and stimulating vapors:

"The first surgical operation during an anæsthetic condition, induced by the inhalation of the fumes of rum, was the reduction of a dislocation of the hip-joint of a negro, 'Bob.' Louisiana. By Dr. Collyer. December, 1839.

"Extraction of tooth from Miss Mary Allen during an insensible condition, induced by the inhalation of ether combined with the fumes from poppy seeds. Philadelphia. By Dr. Collyer. November, 1842.

"Publication of 'Psycography,' (copy-righted work,) wherein, at pages 26, 27 and 28, particular mention is made that the inhalation of narcotic and stimulating vapors will produce the anæsthetic state. Philadelphia. By Dr. Collyer. May, 1843.

"Insensibility produced by the inhalation of protoxide of nitrogen. Hartford, Connecticut. Horace Wells. 1845.

"Publication in Boston *Medical Journal* that ether, combined with opium, would produce the anæsthetic state. Boston. By Dr. Smilie. June, 1846.

"Administration of ether by Drs. Morton and Jackson. Boston, U. S. September, 1846.

"Inhalation of chloroform. Edinburgh. By Dr. Simpson. 1854. (1847?—Z.)

"Amylene. London. By Dr. Snow. 1857.

"Bichloride of methylene. London. By Dr. Richardson. 1867."

